# Laparoscopic surgery for primary ovarian and retroperitoneal hydatid disease: A case report

**DOI:** 10.1097/MD.0000000000009667

**Published:** 2018-01-19

**Authors:** Qinghua Zhao, Jin Luo, Qin Zhang, Tianyan Leng, Lihua Yang

**Affiliations:** Department of Gynecology, the Second Affiliated Hospital of Kunming Medical University, Kunming, China.

**Keywords:** echinococcosis, hydatid disease, laparoscope, ovary, retroperitoneum

## Abstract

**Rationale:**

Cystic echinococcosis (CE) is a parasitic zoonosis caused by echinococcus larvae. Manifestations of the disease include a severe damage to the liver and lung. Damages to the mesentery, omentum, spleen, brain, heart, bone, thyroid, kidney, and uterus are rarely observed. Moreover, primary ovarian and retroperitoneal hydatid disease is extremely rare, and is easily ignored or misdiagnosed.

**Patient concerns:**

We present a case of CE in a 34-year-old female who presented with an adnexal mass detected by B-ultrasound. Adnexal and retroperitoneal masses were removed by laparoscopic surgery. Postoperative pathological report (retroperitoneal cyst) *Echinococcus granulosus*.

**Diagnoses:**

Primary ovarian and retroperitoneal hydatid disease.

**Interventions:**

The patient received intravenous injection of dexamethasone (10 mg) before cyst resection to prevent allergic reactions and oral albendazole (600 mg BID) for 14 days to prevent relapse postsurgery.

**Outcomes:**

The patient revealed no recurrence of disease and no reportable significant changes in 3 months.

**Lessons:**

We present here a case report of CE. This case described herein inhabited a nonendemic region. Gentle and careful operation, and avoiding cyst rupture is the key to insuring success of the surgery. For safety, dexamethasone may be used before cyst resection to prevent anaphylaxis, and mebendazole can be used postoperatively to prevent relapse.

## 1 Introduction

Cystic echinococcosis (CE) is an endemic parasitosis caused by infection of *Echinococcus granulosus* larvae, of which the dog is a dominant carrier.^[1,2]^ Humans are infected following consumption of echinococcus eggs in contaminated food, water or contact with infected animals. Oncospheres that develop into hydatid cysts, provoke severe liver and lung damage,^[3]^ with possible involvement of the intestinal mesentery, spleen, brain, heart, and kidney.

Primary ovarian and retroperitoneal hydatid disease is very rare with few reported cases,^[4–6]^ and is seen in epidemic farming areas where it migrates from endemic to nonendemic regions. Echinococcosis is usually asymptomatic and often associated with occasional dull pain or acute diffuse peritonitis. However, anaphylactic shock, due to cyst rupture, and fluid draining to the abdominal cavity following surgery, are the most severe complications of hydatid disease, which carries a risk of high mortality.

We presented a case without a history of close contact with dogs and lacking clinical symptoms from a nonendemic region. However, on physical examination, a pelvic mass was discovered.

## 2 Case description

A 34-year-old female was admitted following the diagnosis of adnexal mass detection over a 1 year period by B-ultrasound. The patient was born and raised in Lijiang, Yunnan province, which belongs to a nonendemic area. However, during frequent business travels, this patient visited Echinococcosis endemic regions like Sichuan and Guangxi provinces for prolonged periods. Menstrual obstetric history revealed regular menstruation and occasional dysmenorrhea not requiring pain-relieving drugs, good health, G2P1 (i.e., gravida 2, para 1), and a single occasion of induced-abortion 3 years ago.

**Figure 1:**
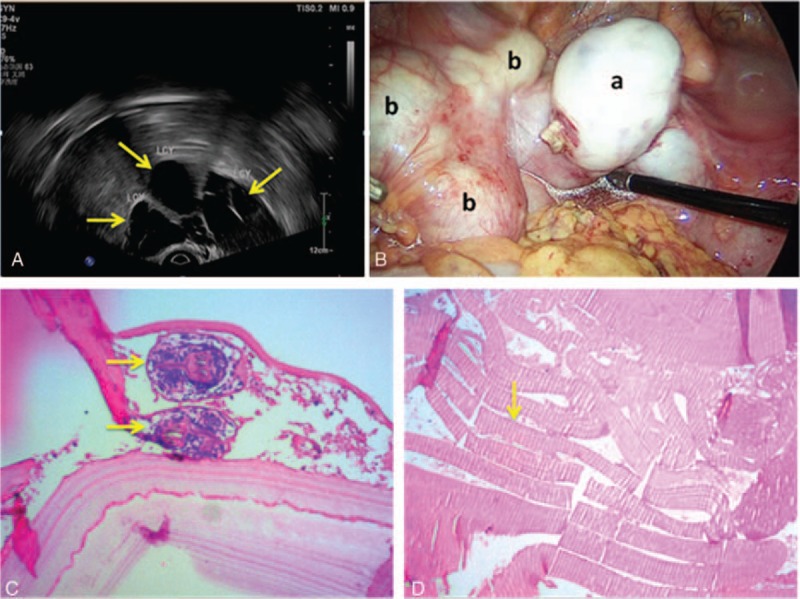
(A) Preoperative vaginal B-ultrasound. Yellow arrows identify multiple left accessory cysts. (B) Laparoscopic surgery (a, left ovarian hydatid cyst; b, retroperitoneal hydatid cyst). (C) HE staining: yellow arrows identify the retroperitoneal hydatid scolex (×100). (D) HE staining: a yellow arrow identifies the left ovarian bursa (×100).

**Figure 2:**
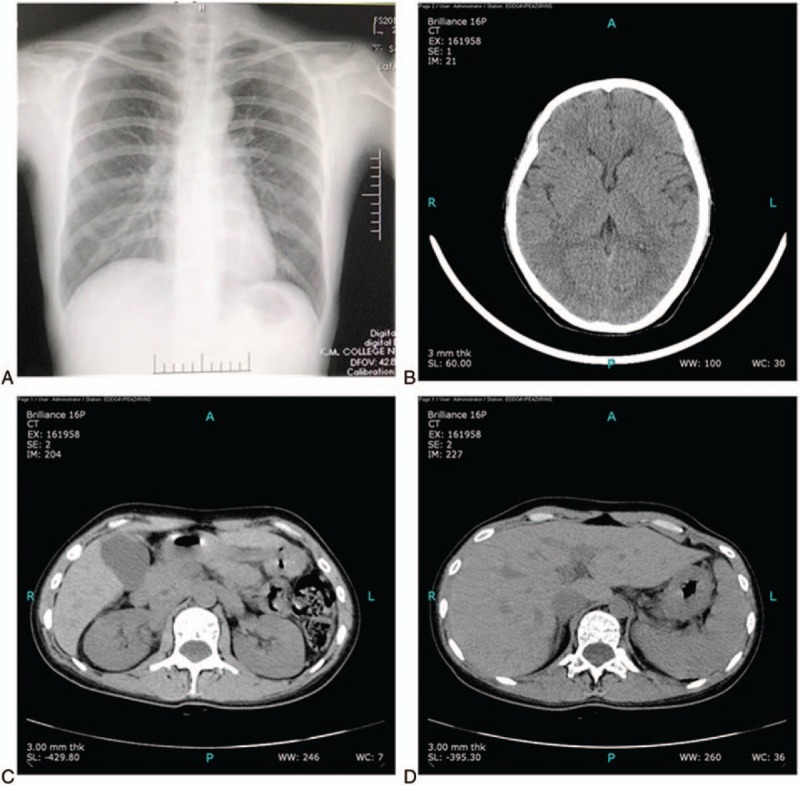
Postoperative CT scan. (A) Chest X-ray was normal. (B) Head CT revealed no abnormalities. (C) Abdominal CT scan of the bilateral kidney was normal. (D) Abdominal CT scan of the left liver lobe showed calcification, and no other abnormalities.

Bilateral annex examination identified a left side solid cystic mass of approximately 8 cm,^[2]^ with a clear border, medium density, poor motion, and mild tenderness. Gynecological vaginal ultrasound detected adenomyosis with the uterus having dimensions of 10.8 × 5.3 × 6.6 cm, and hypoechoic masses of 3.5 × 2.9 cm, 3.4 × 3.0 cm, and 5.5 × 4.5 cm at the left adnexa with internal compartments (features undefined; Fig. 1A). Preoperative routine urine and stool analysis, electrocardiogram, liver and renal function, and chest radiograph showed normal results (Fig. 2A). On November 17, 2016, the patient underwent laparoscopic exploration, and showed omental and abdominal wall patchy adhesions, no pelvic effusion, retroposition of the uterus with an irregular size increase to approximately 10 × 8 × 7 cm, and with anterior wall thickening, a milky white mass with dimensions of 8 × 8 × 7 cm at the left annex, without evidence of neoplasm or rupture, which tightly adhered to the left uterine horn, fallopian tube, pelvic wall, and intestine. Multiple milky white retroperitoneal masses on the left pelvic cavity were visible (Fig. 1B) without abnormalities evident in the appearance of the right fallopian tube and ovary, liver or spleen evident.

Laparoscopic resection of the left side adnexal mass revealed 3 cystic cavities in the mass, which were filled with pale yellow viscous fluid and several blisters of different sizes, on which evidence the possibility of hydatid disease was considered. After opening the peritoneum, a long strip of approximately 10 × 5 × 5 cm of a twisted milk white package was seen, which connected tightly and extended along the left external iliac artery toward the interior to the left ureter. Care was executed to peel the mass off the ureter and blood vessel. In addition, intravenous injection of dexamethasone (10 mg) was administered in advance to prevent allergic reactions.

However, with a tight adhesion to the blood vessels and ureter presented among the mass. Cyst rupture occurred during surgery. Moreover, several compartments were found inside the cyst that were filled with a pale yellow viscous fluid and several blisters of different size were exposed. Postoperative pathological diagnosis confirmed (retroperitoneal cyst) *E granulosus*, which is also known as hydatid disease (Fig. 1C and D). Postoperatively, the patient was closely monitored for vital signs and changes in disease status, which were unremarkable. Examination of CT scans 3 days postsurgery indicated no abnormality in the head, or in the upper, middle, and lower abdomen, or in the pelvic cavity (Fig. 2B–D). The patient recovered well and was discharged 5 days postsurgery, with no adverse visible clinical signs, symptoms, or discomfort. Oral administration of albendazole (600 mg BID) was continued for 14 days. Follow-up examinations at 1 and 3 months following the procedure revealed no recurrence of disease as determined by gynecological transvaginal B-ultrasound, which confirmed normal postoperative menstruation, and no reportable significant changes in the patient.

## 3 Discussion

We presented a 34-year-old female patient who was admitted on diagnosis of an adnexal mass by B-ultrasound. Adnexal and retroperitoneal masses were removed by laparoscopic surgery and the patient was diagnosed as primary ovarian and retroperitoneal hydatid disease by postoperative pathology.

The lessons learned from presurgical indefinite diagnosis of this case include: knowledge that the patient was born and lived for a prolonged period of time in Yunnan—a nonepidemic area for hydatid disease. Additionally, the patient provided no prior history of close contact with dogs or sheep, and frequently travelled on business between epidemic areas of Sichuan and Guangxi provinces that surround Yunnan. Thus, awareness of hydatid disease could be improved. No other symptoms showed out on admission of the patient with a diagnosis with “adnexal masses of more than 1 year via B-ultrasound.” A pelvic mass was also discovered by physical examination. Genital system hydatid disease is rarely seen in gynecology clinics. Thus, expertise in the differential diagnosis of ovarian cyst and pelvic echinococcosis by B-ultrasound might be insufficient.

The key to successful therapy of hydatid cyst lies in protecting the surgical field from error, and preventing dissemination of the vesicular cyst or scolex following leaking of hydatid cyst fluid.^[7]^ All patients should be treated postoperatively with albendazole or its liposomal oral emulsion to prevent recurrence.

The most common complication of hydatid disease is anaphylactic shock from a strong allergic reaction to cystic fluid release in the abdominal cavity caused by surgically induced cyst rupture during surgery or by implementing insufficient protective measures. The current case was not preoperatively diagnosed, and the possibility of hydatid disease was considered only following exploratory surgery. Dexamethasone administration presurgery coupled with abdominal rinsing and irrigation procedures with surgical/sterile saline mitigated para- and postsurgical allergic responsiveness.

The current case is supported by other reports.^[2,8–13]^ Gurdal et al^[8]^ described a 48-year old woman presenting with a 3-month history of right flank pain, but otherwise without remarkable symptoms. However, by abdominal-pelvic ultrasonography (USG), a solid mass of 60 mm with regular borders and evidence of calcification was discovered in the right suprarenal region.^[8]^ By abdominal contrast CT, a 60 × 40 mm solid mass was seen with egg shell calcification of the soft tissue mass. Exploratory surgery revealed a solid immobile mass, which was suprarenally positioned and resected with a segment of the apical pole of the right kidney. Over a 12-month follow-up, the patient was disease free. Similarly a 76-year-old postmenopausal woman was admitted to an emergency unit with a 10-day history of urinary retention.^[9]^ Authors described an unusual presentation of a solid mass with cystic components located anterolaterally to the bladder and pelvis. Pathology confirmed diagnosis of hydatid cyst, and the patient was discharged following uneventful recovery and prescribed oral albendazole (800 mg QD) as adjuvant therapy for 6 months. Similar cases have been reported, wherein differential diagnosis confirmed primary adnexial hydatid cyst that mimicked a multicystic ovarian tumor.^[10]^ Following resection, the patient was discharged and administered mebendazole (100 mg b.i.d.) for 4 months. Similarly, a diagnosis of hydatid cyst of the ovary was discovered incidentally in a 30-year-old female patient following surgery for suspected ovarian cyst. Exploratory laparotomy revealed a marked cystic mass close to the pelvis.^[2]^ Postsurgical antibiotic and steroidal therapy and recovery was uneventful.

Whilst the above cases reported presentation of hydatid cyst in female patients,^[2,8–10]^ there is also evidence of this condition in male subjects.^[2,9–13]^ Differential surgical diagnosis was key, and resection of the cyst avoided potential adverse outcomes of cyst rupture and anaphylaxis.

Patients travelling and carrying slow grow mass in ovarian should consider possibility of hydatid disease. Laparoscopic resection was feasible in primary ovarian and retroperitoneal hydatid disease patient. Gentle and careful operation, and avoiding cyst rupture are the key to insuring success of the surgery. For safety, dexamethasone may be used before cyst resection to prevent anaphylaxis, and mebendazole can be used postoperatively to prevent relapse.

## 4 Author contributions

Conceptualization: Qinghua Zhao and Jin Luo.

Data curation: Qin Zhang and Tianyan Leng.

Formal analysis of the data: Qinghua Zhao, Jin Luo, Qin Zhang, Tianyan Leng, and Lihua Yang.
